# Tumor-stromal crosstalk in pancreatic cancer and tissue fibrosis

**DOI:** 10.1186/s12943-018-0927-5

**Published:** 2019-01-21

**Authors:** Divya Thomas, Prakash Radhakrishnan

**Affiliations:** 1 Eppley Institute for Research in Cancer and Allied Diseases, Fred & Pamela Buffett Cancer Center, University of Nebraska Medical Center, 986805 Nebraska Medical Center, Omaha, NE 68198-6805 USA; 2 Department of Biochemistry and Molecular Biology, University of Nebraska Medical Center, Omaha, NE USA; 3 Department of Pathology and Microbiology, University of Nebraska Medical Center, Omaha, NE USA; 4 Department of Genetics, Cell Biology and Anatomy, University of Nebraska Medical Center, Omaha, NE USA

**Keywords:** Pancreatic Cancer, Desmoplasia, Fibrosis, Stellate cells, Extracellular matrix, Tumor microenvironment

## Abstract

Pancreatic ductal adenocarcinoma (PDAC) is a devastating disease with high morbidity and mortality worldwide. To date, limited therapeutic achievements targeting cell proliferation and related mechanisms has led researchers to focus on the microenvironment where pancreatic cancers develop. The anomalous proliferation of stromal cells, such as pancreatic stellate cells, and an increased deposition of altered matrix proteins create an environment that facilitates tumor growth, metastasis and drug resistance. Here, we summarize our understanding of recent advances in research about the role of fibrosis in pancreatic cancer progression, with particular emphasize on the involvement of fibrotic machineries such as wound healing, extra cellular matrix degradation, and epithelial-to-mesenchymal transition. The precise influence of these mechanisms on the biological behaviors and growth of cancer cells has great impact on clinical therapy and therefore deserves more attention. We also discuss the role of various stromal components in conferring drug resistance to PDAC which further worsening the pessimistic disease prognosis. A more in depth understanding of cancer-stroma crosstalk within the tumor microenvironment and stroma based clinical and translational therapies may provide new therapeutic strategies for the prevention of pancreatic cancer progression.

## Background

According to the American Cancer Society, in the year 2018, an estimated 55,440 people will be diagnosed with and 44,340 will die of pancreatic cancer in United States [1]. The genomic characterization of pancreatic cancer patients reveals the high heterogenicity of this disease [2]. Pancreatic ductal adenocarcinoma (PDAC) is projected by researchers to become the second-most leading cause of cancer-related death in the US by 2030 [3]. The limited availability of diagnostic approaches, and surgery as the solely existing curative option with the survival possibility of only 10% of diagnostic patients, increases the dreadfulness of this disease [4]. Though research advancement in imaging techniques and the use of certain biomarkers improves identification of biological compounds that target specific signaling cascades to extend the overall survival of patients, metastasis remains an obstacle for clinicians and researchers [5]. Several genetic and epigenetic research studies have identified key genetic alterations responsible for the development of PDAC, including mutation in Kras [6, 7], p53 [8], BRCA1 and BRCA2 [9], and SMAD4 [10]. However, targeting these genetic or epigenetic variations has yet to produce a useful clinical therapeutic against PDAC. There is a critical need at this juncture for new strategies to prevent pancreatic cancer progression and metastasis.

Tissue fibrosis as a trigger for cancer formation and metastasis was initially identified in the early 1950s [11, 12]. Fibrosis represents a pathological condition characterized by the infiltration and proliferation of mesenchymal cells in the interstitial space, which occurs as a result of injuries to the epithelial cells and ultimately results in organ dysfunction. Uncontrolled wound repair mechanisms and aberrant inflammatory responses are believed to trigger organ fibrosis [13]. Matrix remodeling, a crucial mechanism for the repair process, is found to be dysregulated during fibrotic machinery. The fibril organization of the extra cellular matrix (ECM) facilitates production of pro-fibrotic cytokines and growth factors that results in permanent scar formation in the organ [14]. Because it is the regulator of various cellular behaviors and mediator of cellular communications, any perturbations in the matrix architecture highly influences the proliferation and migration of cells [15]. Such abnormal proliferation of stromal cells, along with aberrated ECM dynamics, promotes formation of a tumorigenic microenvironment that leads to malignant transformation, and facilitates the ability of cancer cells to survive and invade [16]. Therefore, tumorigenesis and cancer metastasis are highly influenced by an altered ECM that usually occurs as a result of an abortive attempt to repair injured tissue. In this review, we bring together the emerging aspects of tumor-stromal interactions in the microenvironment, organ fibrosis and pancreatic cancer metastasis to identify challenges in designing novel therapeutic strategies to intervene in the progression of pancreatic cancer.

### The tumor microenvironment of pancreatic cancer: Altered extracellular matrix alliances fibrosis and cancer

The tissue microenvironment comprises an active population of cellular and non-cellular components that forms an organized niche to regulate the homeostasis of any organ [17, 18]. Over the past few decades, significant understanding has been achieved in identifying several oncogenes and tumor suppressor genes in pancreatic cancer. These genes regulate cell growth, inflammation, apoptosis, and multifaceted signaling networks [19, 20]. For instance, in pancreatic cancer, Kras mutations are predominant and drive tumorigenesis. Several other genes, such as CDKN2A, TP53 and SMAD4, participate in the progression of cancer. Accumulation of such mutations in the normal cell drive it to a benign tumor state and stays dormant while it lacks the ability to invade and metastasize other parts and form vasculature [21, 22]. A very large body of evidence suggests the involvement of aforementioned genes and tumor microenvironments contributing to PC progression. However, the mechanism of tumor microenvironment mediated progression of pancreatic cancer remains elusive.

How tumor cells communicate with external signals from neighboring cells are what make it so perilous, and we must address the fact that tumor expansion greatly depends on its microenvironment. The tumor microenvironment consists of not only tumor cells, but also other cell types such as fibroblasts, immune cells (T & B Cells, NK cells, Tumor associated macrophages), blood vessels, ECM and other signaling molecules [23, 24]. The cellular and non-cellular components in the microenvironment maintain tissue homeostasis and play a pivotal role in opposing resistance to malignant cell growth [25]. Three different types of normal cells in the tumor microenvironment have been identified: stromal cells, fibroblasts, and immune cells. Cellular cross-talk between cancer and stromal cells is critical for cancer progression [26]. Fibroblasts and stromal cells secrete growth factors, such as fibroblast growth factor (FGF), to mediate tumor progression. Apart from tumor progression and support to malignant cells, these cell types function as chemoattractant that instigate migration of other cells to the microenvironment. The pancreatic tumor microenvironment consists of abundant stromal cells, especially pancreatic stellate cells [27], and tumor stroma possess dual signals of tumor suppression and promotion. For instance, inhibition of Hedgehog signaling reduced the abundance of stroma and improved vascular delivery of the anticancer agent, gemcitabine [28, 29]. ECM is yet another major factor in the progression of PDAC. ECM contains proteins such as laminin, fibronectin, proteoglycans, glycoproteins, and polysaccharides, with diverse physical and biochemical properties. Collagens are the most abundant matrix proteins; wherein pancreatic tissue undergoes both synthetic and degradative processes. Furthermore, collagen architecture needs to be maintained in normal homeostasis. Any abrupt change in the deposition of collagens results in tissue fibrosis, thereby limiting tissue function [30, 31]. Cancer-associated fibroblasts (CAFs) are vital components of the tumor microenvironment involved in the stromal-to-tumor interaction [32].

**Fig. 1:**
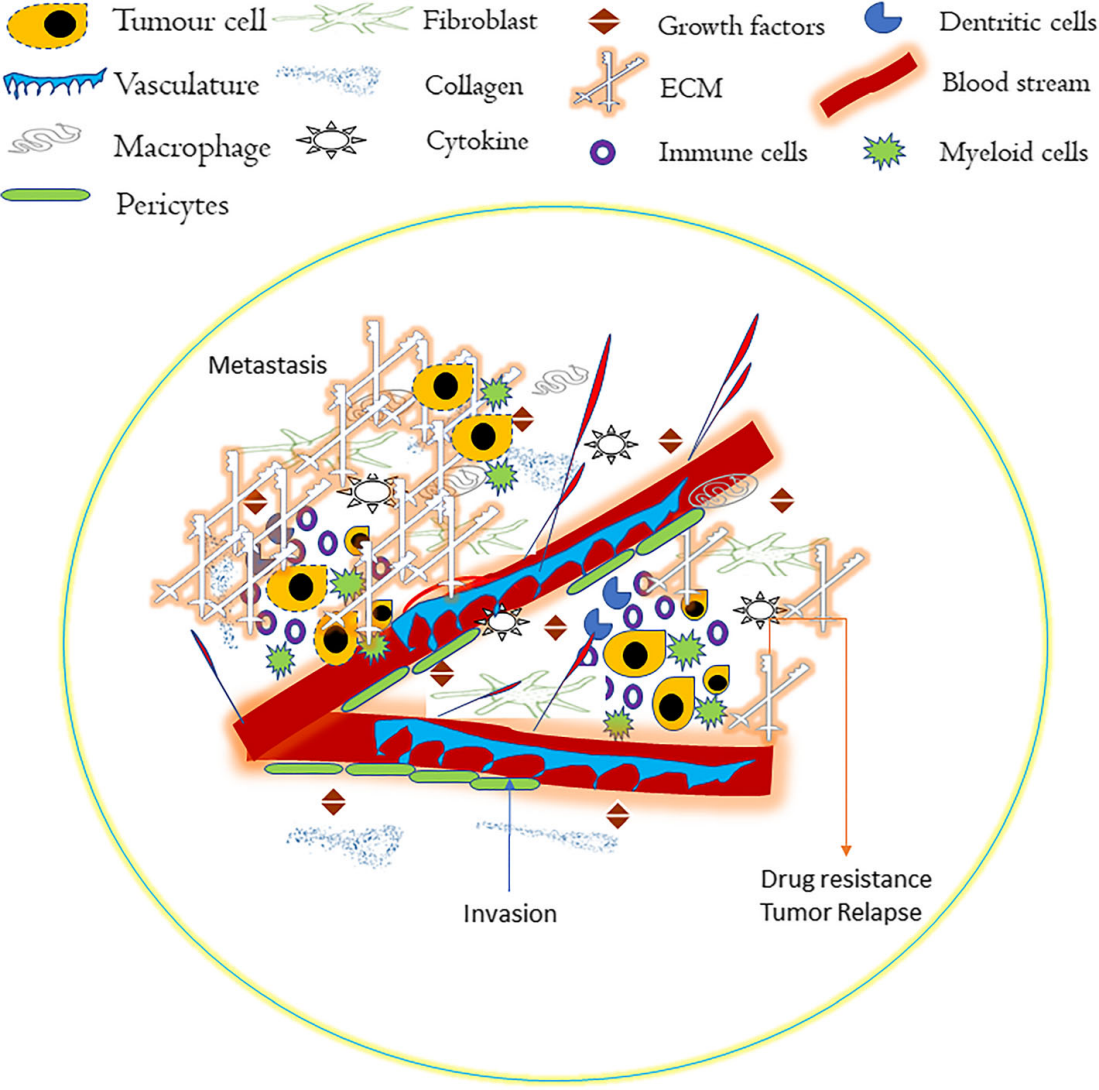
Tumor microenvironment in pancreatic cancer: Formation and expansion of tumor mass confined by the altered basement membrane and stroma causes collapse in blood vessels. In turn, the recruitment of inflammatory and immune cells such as cytokines, macrophages, dendritic cells and growth factors contribute in the remodeling of altered extracellular matrix. These pathological events together contribute to tumor growth, invasion, metastasis and drug resistance

An orchestrated crosstalk between mutated cells and the microenvironment leads to altered ECM composition and results in activation of resident fibroblasts and recruitment of inflammatory cells and pericytes, thereby promoting angiogenesis and cancer progression (Fig. 1). It is worthwhile to address: a. the specific signals that cancer cells transmit and receive from their environment; and b. how the received signals initiate a malignant growth. Recently, using the emerging orthotopic tumor model, which mimics the effects of tumor microenvironments, rather than ectopic transplantation models, a connection was reported of the pancreatic tumor microenvironment and associated cells [33]. Nevertheless, these questions remain unanswered, but further attempts to understand these findings will, we believe open the way toward developing a novel strategy to inhibit PDAC progression.

### Fibrosis in pancreatic ductal adenocarcinoma

A dense fibrotic stroma or desmoplasia, comprises excessive production of ECM and proliferation of stromal cells, is found with both chronic pancreatitis and PDAC. The replacement of normal parenchyma with a desmoplastic environment results in exocrine and endocrine inefficiency of pancreas and is one of the important pathophysiological hallmarks of PDAC. Of interest, when compared with other solid tumors, PDAC is reported to have the highest amount of stroma [34]. The desmoplasia imposes a high burden during radiotherapy, given that it is very difficult to discriminate between tumor growth and fibrotic stroma. Desmoplastic reaction is characterized by fibrillar proteins, glycoproteins, proliferating fibroblasts, inflammatory cytokines, and immune cells. This desmoplastic reaction in PDAC is responsible for increased drug resistance [35]. Several signaling cascades that affect tumor epithelial and stromal compartments have been reported to play critical role in the pathogenesis of PDAC in connection with fibrotic responses. While Kras mutation is an early event in tumorigenesis that occurs in the pancreatic epithelial compartment, transforming growth factor (TGF)-β signaling is known as a dual compartment signaling cascade with a prominent function in the epithelial and stromal compartments [36]. The composition of fibrogenic mediators in pancreatic fibrosis was demonstrated in a TGF-β over-expressed transgenic mouse model, and the level of collagen I and III were increased initially, followed by increased deposition of fibronectin, fibroblast growth factors, and pancreatic stellate cells. Furthermore, pancreatic ECM machinery is mainly regulated through TGF-β signaling [37]. Recently, a research group reported that TGF-β within the stromal compartment of the pancreas directly participates in cancer progression and metastasis, using a monoclonal antibody, G28, which neutralizes the activity of murine TGF-βII. The resulting decreased activation of fibroblasts and collagen deposition when using G28 strongly support the significance of TGF-β-mediated desmoplastic reaction during pancreatic cancer progression [38]. An interesting and significant role of TGF-β as a tumor suppressor in the early stage of PDAC, through promotion of apoptosis and cell cycle arrest at the G1 phase, has also been reported [39, 40], and the function of TGF-β signaling in PDAC appears to be complex. However, it has been more evidently demonstrated that the decisive role of the tumor microenvironment obtains in the action of TGF-β as a tumor suppressor or promoter. Pancreatic stellate cells in the stromal compartment are responsible for excess matrix production, and, being a potent activator of stellate cells, TGF- β accelerates the metastasis of cancer cells in the stromal region [41]. Altered expression of TGF- β in the pancreatic stroma results in the dysregulated turnover of ECM and leads to aberrant accumulation of matrix proteins and immune cells, which further creates a favorable environment for invasion and metastasis of cancer cells.

**Fig. 2:**
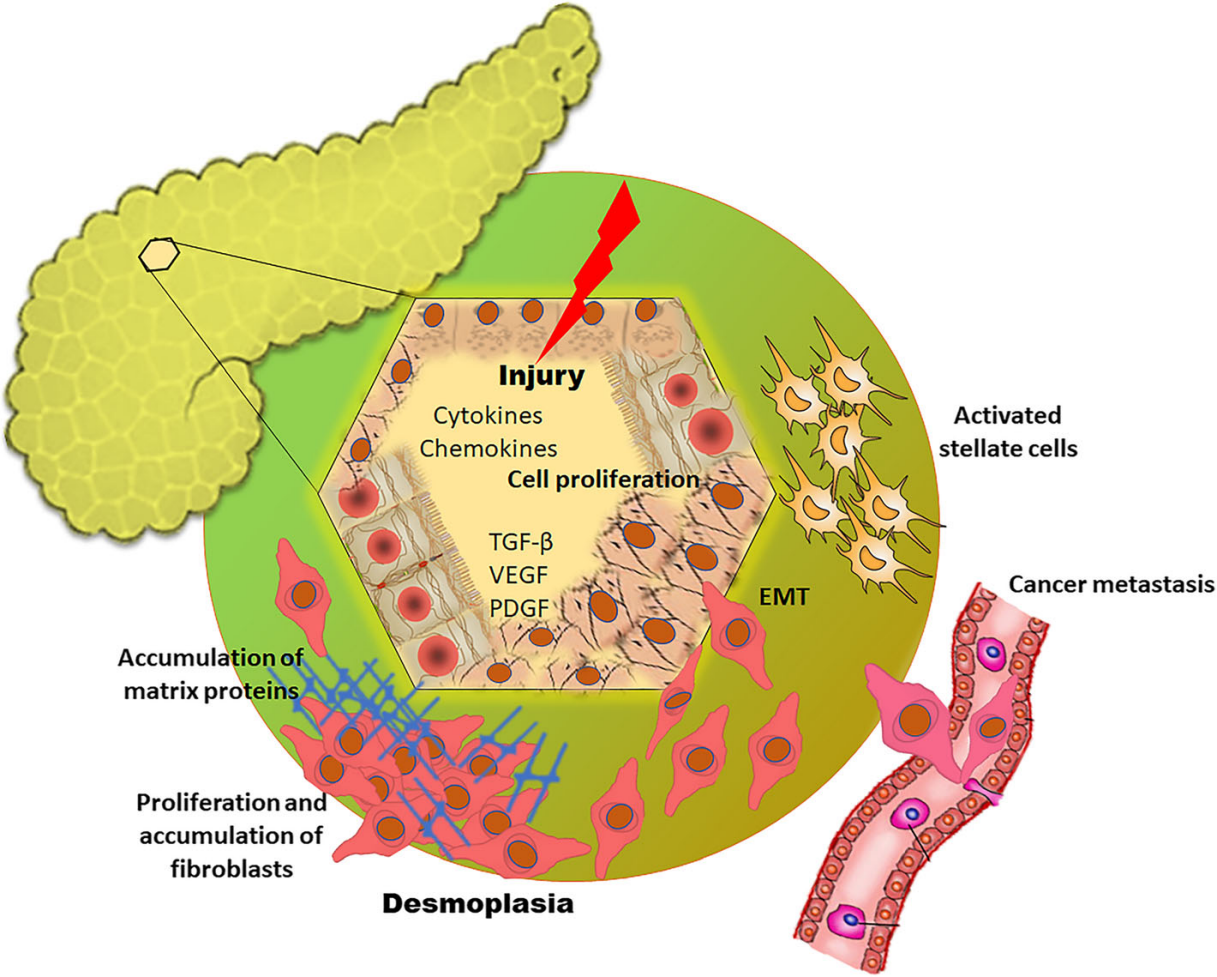
Possible mechanisms involved in pancreatic cancer metastasis and fibrosis: Perpetuation of pancreatic stellate cells activation by cytokines/chemokines/growth factors mediated mechanisms results in pancreatic fibrosis with aberrant desmoplasia. Alterations in the epithelial cell phenotype, or EMT, may be driven by tumorigenic signal pathways that are consequently activated during desmoplastic reactions. In response to EMT driving signals, tumor cells in the invasive front enter into the circulation and metastasize to distant sites

Fibrogenesis in the pancreas itself is a complex process which occurs due to multiple factors, such as external injury to the tissue, chronic pancreatitis, and aberrant cell death. Such tissue injury activates the release of several inflammatory cytokines, chemokines, and growth factors to the site of injury [42]. These growth factors, including TGF-β, vascular endothelial growth factor (VEGF), platelet derived growth factor (PDGF), angiotensin, and others, activate pancreatic stellate cells that facilitate the accumulation of myofibroblasts. The proliferation of fibroblasts and conversion of fibroblasts to myofibroblasts are the key events responsible for the excessive deposition of ECM proteins, particularly collagen [43, 44]. The resulting collagen-rich meshwork creates a favorable environment for survival and proliferation of cancer cells [45]. In addition to TGF-β, other signaling pathways such as VEGF and PDGF also play important roles in triggering desmoplastic reaction in the stromal compartment of the pancreas [46, 47]. External or internal injury to the epithelial cells, as well as activated pancreatic stellate cells, are also shown to activate cell proliferation by undergoing epithelial-to-mesenchymal transition (EMT), a mechanism which further links fibrosis and cancer metastasis [48] (Fig. 2). Interestingly, parallel biological reactions controlled by the same set of molecules are involved in pancreatic fibrosis and cancer progression. It is hoped that, the increasing evidence of fibroblasts and stromal elements in the pancreas will lead to a paradigm shift in the treatment modalities of pancreatic cancer.

### Pancreatic stellate cells as sprouted seeds for tumor progression

Although active research in pancreatic cancer is ongoing, it has yet to develop a targeted therapy and has failed to prolong the survival of patients with PDAC. This situation demands more research focus on the infrastructure of tumor, so called stroma, as it is believed that desmoplastic reactions and fibrotic stroma protect the cancer cell as a barrier from chemotherapy [49]. Activated pancreatic stellate cells (PSCs) in the stroma, present as a quiescent or inactive form in normal pancreatic tissue, are considered as the sprouted seed of cancer progression and metastasis. Although as early as 1876, the star-shaped lipid containing cells was initially identified in the liver [50], the first description of these cells in the pancreas was not made until 1982 [51]. In the parenchyma of normal pancreas, PSCs exist in an inactivated form that contains abundant vitamin A and fat droplets in its cytoplasm. Loss of lipid droplets by any physiological or pathological reactions results in the activation of PSCs, which show increased expression of α-smooth muscle actin (α-SMA) and desmin and fibroblast-like features [52]. PSCs are highly responsible for the aberrant secretion of matrix proteins such as collagen, laminin, and fibronectin in the ECM that induces desmoplastic reactions in the pancreas [53, 54]. The normal function of PSCs in the healthy pancreas includes immune reaction, phagocytosis, and stimulation of amylase secretion from the pancreas [55]. However, tumorigenesis induces conformational change in PSCs, which then exhibit myofibroblast-like characteristics and have a strong effect upon tumor progression and metastasis. Fibroblast-like cells expressing α-SMA, collagen I and III, laminin, and fibronectin have been identified in the fibrotic area in pancreatic cancer tissue by electron microscopy and immunofluorescence analysis [56]. As the balance regulator in ECM by way of secreting matrix metalloproteases and tissue inhibitors of matrix metalloproteases, PSCs highly contribute to fibrotic formation in the pancreas. These cells also stimulate angiogenesis and facilitate the invasion and extravasation of cancer cells. A fluorescent, in situ hybridization study revealed that human PSCs accompany cancer cells to various metastatic sites and facilitate intravasation and extravasation of cells from the blood vessel [57]. PSCs also promote EMT, which is seen by the decreased expression of epithelial marker E-cadherin and increased expression of mesenchymal markers such as N-cadherin, vimentin, and transcriptional factor Snail [31]. PSCs are highly activated in hypoxic condition, and, since pancreatic cancers are hypovascular, PSCs are believed to be the triggering factor for cancer cell invasion and metastasis [58]. In this view, PSCs act as the sprouting seed for progression of the tumor tree, with much evidence pointing to its crucial role in inducing fibrotic environment that facilitates cancer metastasis.

### Blood coagulation cascade, hypoxia and wound healing: The seed bomb of tumor progression

It is interesting to discuss the interrelationship between cancer progression and the blood coagulation cascade, wound healing machinery, and fibrotic reactions. This cross-connection between these biological phenomena was identified over a century ago, when Dvorak called cancer ‘wounds that do not heal’ [59]. Cancer usually develops at the site of chronic tissue injury [60], where tissue reactions in response to injury accompany inflammatory reactions, cell proliferation, and tissue re-modeling. In addition, the adequate formation of a hemostatic clot is crucial in healing of normal injury and tissue repair. From this point of view, it is very difficult to separate the blood coagulation cascade from the wound repairing mechanisms [61]. Several research studies support tumorigenesis upon chronic injury to the respective tissues. For example, epidemiological studies show that the risk factor for developing pancreatic cancer is 18.5-fold higher in patients with chronic pancreatitis compared to people with normal pancreas [62]. Further, the critical influence of the coagulation cascade in the inflammatory and fibro-proliferative reactions followed by tissue injury has been extensively studied [63]. Upon injury, injured tissue is covered by a new epithelial layer, which is promoted by the combined action of hematopoietic clot formation, fibroblasts proliferation, and the action of inflammatory cytokines and growth factors. Highly activated platelets are recruited to the site of injury, where serum fibrinogen influences fibrin clot formation, and this reaction induces the formation of new blood vessels. During these mechanisms, activated fibroblasts migrate to the wound bed and stimulate secretion of collagen [64]. However, this collective action can enhance the risk of tumorigenesis in tissues when excessive, and therefore aberrant wound healing can be considered as an initiation event of pancreatic cancer development.

Interestingly, environmental alterations strongly facilitate aberrant wound healing and tissue scar formation; one such key determinant of the tumor microenvironment is tissue oxygenation. Following tissue injury, the compromised blood vessel function leads to a hypoxic state which is sustained further due to the high oxygen demand for the influx of inflammatory cytokines and chemokines [65]. Hypoxia induces several changes in cancer cell metabolism that results in the physiochemical changes in the tumor microenvironment such as reduced pH and increased production of ROS. Similarly, hypoxia promote molecular changes also in normal and cancerous cells by eventually activating the transcription factor hypoxia-inducible factors (HIF) [66]. Hypoxia induced events in the tumor microenvironment are mainly regulated by HIF. Upon tissue injury, the circulation of angiogenic cells such as endothelial precursor cells and mesenchymal stem cells for the appropriate angiogenic responses and wound contraction are mediated through HIF. Consequently, reduced activity of HIF-1α is sufficient to impair wound healing process that results in tissue scar formation [67]. Overexpression of HIF-1α correlates to the activation of pancreatic stellate cells through macrophage recruitment which is critical for the development of desmoplasia has been reported [68]. Hypoxic state induces the production of cytokines and chemokines such as CCL2 and its hematopoietic cell receptor CC chemokine receptor 2 (CCR2), VEGF-1, chemokine (C-X3-C motif) receptor 1 (CX3CR1) to attract macrophages and monocytes in the microenvironment. Interaction between these macrophages and pancreatic stellate cells promote the formation of dense desmoplasia around the tumor [69]. Macrophages are functioning in the activation of stellate cells through the production of several growth factors (TGF-β1, PDGF, fibroblast growth factor-2), cytokines (TNF-α, IL-1, IL-6) and chemokines. CCL2 is a classical chemokine that is highly expressed in the hypoxia region; however, its expression and the severity of hypoxia is still controversial. A research group has reported that hypoxia significantly inhibits the production of CCL2 by macrophages and negatively regulates the macrophage infiltration in pathological tissues [70]. In contrast to this, HIF 1-α promoted the secretion of CCL2 to recruit macrophages which facilitate the formation of pancreatic fibrosis during PDAC progression has also been reported [68]. Promoter region of CCL2 contains hypoxia response elements (HREs) which can bind HIF-1 further support the regulation of CCL2 function by hypoxia [71]. Notably, CCL2 is believed to be the most specific cytokine during monocyte and macrophage recruitment to the wound site. However, other C-C binding proteins such as CCL5 and macrophage inflammatory protein-1 are considered as dispensable for wound repairing mechanisms [72]. Hypoxia promote the production of large amount of CCL2 which is 30 times higher than secreted under normal condition [73]. An impaired macrophage invasion and delayed wound healing in CCL2-null mice effectively demonstrate its importance in wound repairing machinery [74]. Together, both early and late stage of normal wound healing, starting from the angiogenic response, cellular infiltrations, and fibroblasts proliferation to tissue growth and remodeling, is about connected to hypoxia. Targeting hypoxia and angiogenic responses likely to improve the current clinical management of fibrotic conditions during PDAC progression. Yet another hypothesis suggests that repeated injury to pancreatic tissue and regeneration to repair damaged epithelium increases the possibility for somatic mutations. One study reported that chronic pancreatitis in adult mice leads to pancreatic cancer with a mutation in Kras oncogene [75]. Cells with mutated DNA support the initiation and development of desmoplastic reactions in the site of injury and facilitate cancer metastasis through the induction of EMT [76]. However, future studies are warranted to obtain a clearer of the mechanisms of the wound healing machinery and how hypoxia and angiogenic response influence it to induce desmoplastic reactions and thereby tumor progression in the pancreas.

### Chronic inflammation fertilizes tumor progression

In 1863, Rudolf Virchow and his group observed the presence of leukocytes in neoplastic tissue and reported for the first time the possibility of tumorigenesis in areas of chronic inflammation [77]. The immune system and immune response reactions are necessary to maintain healthy surveillance of normal tissues. The escape of unbridled tumor cells within this machinery and upon malignant transformation leads to the creation of a pro-tumorigenic environment [78]. During the early generation of tumor cells in a tissue, immune cells, particularly T cells, detect danger signals from transformed cancerous cells, and in turn initiates immune reactions to eliminate the tumor cells. Hiroaka et al. have reported the anti-tumor response of immune surveillance reactions in the initial intraepithelial phase of human PDAC [79]. However, abundant infiltration of immune cells along with inflammatory cytokines fertilizes the tumor microenvironment. The growth factors, cytokines, and angiogenic mediator-releasing ability of stromal cells in the pancreatic desmoplastic region influences tumor growth to a greater extent [80].

Increased T cell infiltration at the tumor margin has been demonstrated in a genetically engineered mouse model of pancreatic cancer. The exhaustion of T cells from one state to a differential state induces the up-regulation of inhibitory receptors. The development of a special class of antibody checkpoint inhibitors against this T-cell exhaustion provides new hope to overcome drug resistance in PDAC [81]. In yet another study, it has been demonstrated that leukocyte infiltration, regulatory T cells, and tumor-associated macrophages were dominant in the early phase of tumor progression and persisted in invasive stages of tumors in a genetically defined mouse model of PDAC [82]. It has been reported that independent of sex, age, and geographic location, the risk of cancer development is significantly high in patients with chronic pancreatitis, as shown in a study of 2015 human subjects [83]. In a mouse model of PDAC, it was reported that blockade of toll-like receptor (TLR) protects against tumorigenesis through NF-κB, MAPK pathway [84]. Ruxolitinib, a Janus kinase 1 (JAK1)/JAK2 inhibitor, along with capecitabine, is in phase III clinical trials against advanced progression of metastatic pancreatic cancer [85]. Yet another important signaling pathway activated by inflammatory reaction in the pancreas is TGF-β. Aoyagi et al. have reported that overexpression of TGF-β enhances the level of type I and III collagen, and that the mRNA expression of TGF-β, fibroblast growth factor, platelet-derived growth factor A and C, and epidermal growth factor were significantly higher in surgical cancer nodules of pancreatic cancer patients [86]. All these signaling transcription factors further facilitate the secretion of inflammatory cells and thus create a favorable microenvironment with growth factors, reactive oxygen species, and inflammatory cells that have the potential to proliferate tumor cells. These pathological factors potentiate tumor growth by inducing more proliferation of fibroblasts in the desmoplasia, greater stimulation of angiogenesis, and further enabling of metastatic spread of tumors to distant organs. In this way, the journey of tumor cells from tumorigenesis to metastasis is fertilized by inflammation and fibrotic reactions.

### Extracellular matrix: The fertile soil for tumor progression

**Fig. 3:**
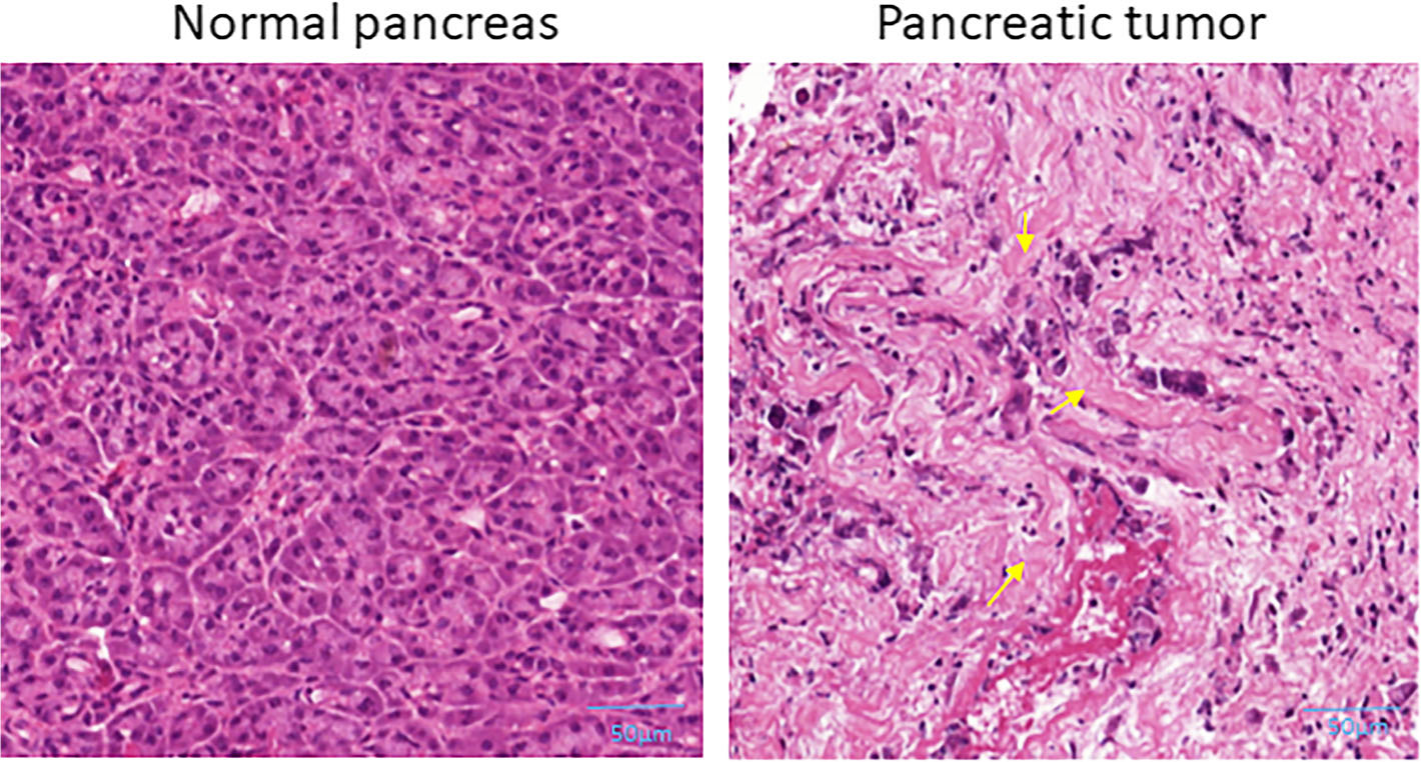
Histopathological staining of normal and primary pancreas tumor tissues. Development of highly dense fibrotic (Desmoplasia) environment around the tumor cells was detected (yellow color arrows) in the poorly differentiated (stage IV) PDAC tumor tissue but not in the normal pancreas tissue (scale bar: 50 μm). The normal and pancreatic tumor tissues were procured from the UNMC Rapid Autopsy Pancreas Tumor tissue bank

The extra cellular matrix (ECM) is a complex network of macromolecules that provides a physical scaffold to maintain tissue architecture. ECM not only provides structural foundation but generates various biochemical signals that regulate cellular behavior and function [87]. ECM plays a major role in tumor progression, given that it compasses 70% of the tumor microenvironment. All the proteins, glycoproteins, and polysaccharide components of ECM are generally produced by epithelial and stromal cells whose purpose is to separate the epithelial compartment from the stromal compartment. Collagen, laminin, and fibronectin meshwork form the basement membrane [88]. The altered organization and enhanced post-translational modification of matrix proteins results in the induction of desmoplasia (Fig. 3); and this fibrotic area accounts for 90% of the tumor area in the pancreas [89]. The tumorigenic functions of major proteins in the ECM is discussed in Table 1. In fact, the frequency and features of the tumor are designed by the stromal cells that surround it. This hypothesis is strongly supported by the findings of Hwang et al., who have reported that co-injection of pancreatic tumor cells (BxPC3 and Panc1) along with human PSCs in an orthotopic model of pancreatic cancer resulted in increased tumor size and metastasis, all of which are directly proportional with the volume of human PSCs [90]. Kamphorst et al. have reported yet another interesting role of ECM during PDAC progression. The levels of metabolomic parameters, such as glucose, glutamine, serine, creatine phosphate, and upper glycolytic intermediates are very low in tumor tissue compared with neighboring tissue. However, the group has observed an accumulation of essential amino acids in the tumor tissue. Their findings suggest that accumulation of essential amino acids could arise from degradation of extracellular proteins through macropinocytosis [91]. This study was supported by yet another recent work, which showed protein catabolism and macropinocytosis by in situ PDAC cells [92]. They have reported that when there is a lack of glucose or glutamine, the amino acid proline, derived from ECM, acts as a readily available substrate to help tumor cells to compensate for the metabolic challenge [93]. In this way, ECM can be considered to readily facilitate the growth and survival of tumor cells.

**Table 1:** Extracellular matrix components and their functions in pancreatic cancer progression

ECM proteins	Function	Ref
Type 1 collagen	Maintain the invasive phenotype of tumor cells; contributes to drug resistance in PDAC cells by increasing the expression of membrane type 1-MMP which potentiates integrin signaling.	[128, 129]
Hyaluronan	Enhance cell proliferation, metastasis and angiogenesis by interacting with CD44 and receptor for HA-mediated motility (RHAMM)	[130, 131]
Fibronectin	Promote tumor cell survival through increase ROS; Enhancing tumor cell migration through promoting FAK-dependent activation of Rho	[132, 133]
Laminin	Promote metastasis through the formation of hemidesmosomes; cleaved laminin stimulates motility of epithelial cells	[134]
Cytokeratin	Regulate cancer cell growth and motility through modulation of PI3K/Akt signaling	[135, 136]
Osteopontin	Contribute to chemoresistance towards Gemcitabine treatment via activation of NF-κB pathway	[137]
Thrombospondin-1	Facilitate tumor cell motility and metastasis through the up-regulation of matrix metalloprotease 9	[138]
Periostin	Promote cell proliferation, migration and invasion of pancreatic cancer cells	[139]
Versican	Facilitate tumor cell growth and angiogenesis	[140]
Tenascin C	Enhances tumor cell motility through the activation of integrin signaling	[141]

### Epithelial-to-mesenchymal transition facilitates branching out of cancer metastasis

Epithelial-to-mesenchymal transition (EMT) is a well-composed biological program that functions in the framework of embryogenesis, wound healing and repair mechanisms, and cancer metastasis, that drives epithelial cells toward a mesenchymal cell state. Type 1 EMT allows epithelial cells to attain the features of mesenchymal cells with the potential to form secondary epithelia by undergoing the reverse mechanism, called mesenchymal-epithelial transition (MET). Type 1 EMT mainly operates in the context of embryogenesis and organ formation. Type 2 EMT is strongly associated with tissue regeneration, wound healing, and fibrotic machinery. Cancerous cells undergoing type 3 EMT which allow the cells to invade the blood stream and generate cancerous nodules in distant organs, and thereby facilitate metastasis [94]. During EMT, epithelial cells lose their characteristic sheet-like structure by losing cell adherens and tight junction molecules, and acquire mesenchymal features with spindle-shape morphology, migratory capacity, and invasiveness [95]. Cell adherent molecules such as E-cadherin and integrins, and tight junction molecules such as occludenes and claudins, are the major epithelial markers. N-cadherin, Snail, slug, fibronectin, and vimentin are the observed mesenchymal markers [96]. There has been a tremendous increase in recent research trends to monitor the cadherin switch from E-cadherin to N-cadherin as a marker for studying cancer metastasis [97]. Another interesting aspect of EMT phenomena is that of the partial EMT, wherein epithelial cells lose E-cadherin and do not switch to express N-cadherin. This results in an intermediate phase, and this hybrid phenotype is thought to enhance migratory properties and thereby generate circulating tumor cells [98].

**Fig. 4:**
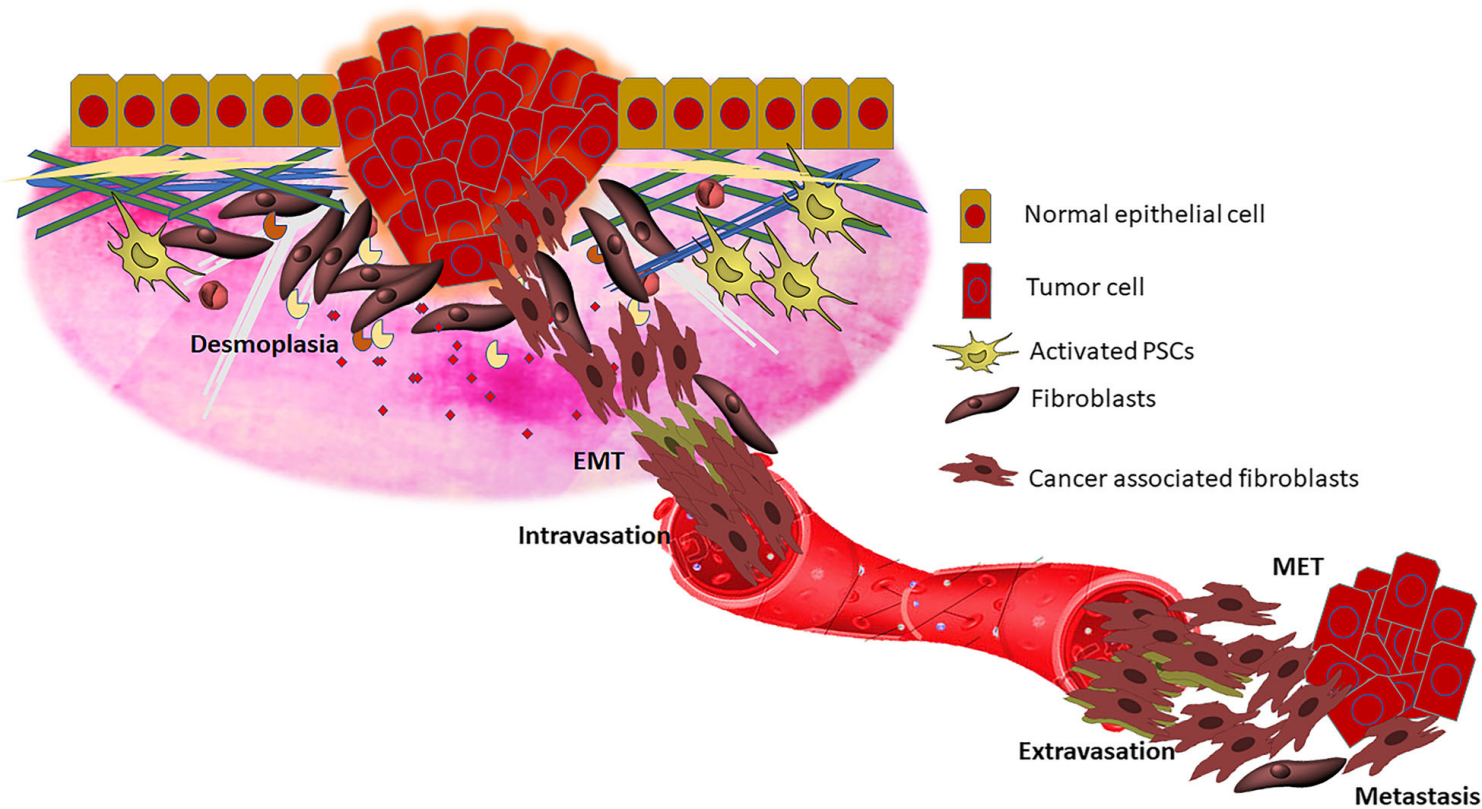
Role of EMT in pancreatic cancer metastasis. During EMT, tumorous epithelial cells undergo various bio-physiological modifications whereby lose their polarity, detach from the basement membrane and invade the surrounding tissue. The angiogenic switch and tumor vasculature facilitate the intravasation of metastatic tumor cells. The cells that survive in circulation extravasate into distant organ and undergo mesenchymal to epithelial transition to form tumor colonization

EMT associated with pancreatic cancer metastasis has been highly influenced by several secretory factors, including TGF-β, PDGF, and VEGF in tumor stroma. TGF-β interacts with other growth factors to stimulate the malignant transformation of epithelial and stromal cells, and activates the proliferation of cancer-associated fibroblasts, thereby inducing fibrotic conditions in the stroma. By this mechanism, pancreatic fibrosis and cancer meet together in the stromal compartment of the pancreas [99, 100]. The plasticity of newly transformed mesenchymal cells from epithelial cells is regulated by various epigenetic factors. The circulating tumor cells are highly ‘metastable,’ with the potential to undergo EMT to form mesenchymal cells or to reverse the mechanism (Fig. 4) [101]. Other than epigenetic factors, several other environmental factors also affect induction of EMT in PDAC. Very recently, Chen et al. reported that hypoxia is a major factor in determining whether the cancerous cells undergo EMT or MET in pancreatic cancerous tissue. BxPc-3 and Panc-1 cells grown under hypoxic conditions showed an increased induction of partial EMT, increased migratory capacity, and invasiveness. Administration of specific inhibitors to HIF1-α significantly reversed these changes in cancer cells [102]. The pro-apoptotic protein Par-4 is reported to have the ability to regulate E-cadherin expression by modulating the promoter activity of transcriptional factor twist and induces MET in metastatic pancreatic cancer cells [103]. Yet another deleterious pathological role of EMT in PDAC is drug resistance [104, 105], in which cancer cells avoid the effects of drugs with the aid of EMT and stem cell properties. The dissemination and stemness of cancerous cells are crucial for the progression of PDAC, therefore a better understanding of how EMT relates to metastasis in PDAC provides new possibilities for diagnostic and therapeutic treatment especially for the metastatic stages of PDAC.

### Drug resistance in pancreatic cancer

Drug resistance in cancer imposes a frustrating problem in cancer therapy. Many cancers are vulnerable to chemotherapy; however, over a certain period, cells develop resistance through mutations in DNA and metabolic changes, and thereafter inhibiting drug effectiveness or showing lack of response [106]. Cancer drug resistance is caused by drug inactivation, aberrant drug efflux, altered DNA repair mechanism, inhibition of cell death, altered EMT, epigenetic factors, or a combination of these processes. Drug resistance in tumor cells exhibits cancer recurrence. Overall, the mechanisms of cancer drug resistance are still not completely understood, though the involvement of multiple signaling pathways have been reported [107, 108]. Many chemotherapeutic drugs, including Gemcitabine, Pimasertib, the Folfirinox regimen (5-fluorouracil [5-FU], irinotecan, oxaliplatin and leucovorin), and Abraxane have been proposed for pancreatic cancer [109–111], and recent reports of the mechanisms of chemoresistance in pancreatic cancer are reviewed [112].

Gemcitabine (2′,2′-difluoro – 2’deoxycytidine), for example, is a deoxycytidine analog and the first line of drugs conventionally used for pancreatic cancer patients. Gemcitabine incorporates replicating DNA and inhibits synthesis of DNA. Gemcitabine uptake is facilitated by nuclear transporters (hENT1), and once inside cells, deoxycytidine kinase (dCK) is the first enzyme involved in the phosphorylation cascade, where it facilitates activation of gemcitabine into gemcitabine di and triphosphates, mediating its cytotoxicity [113]. The metabolites of gemcitabine inhibit other key enzymes, such as cytidine triphosphate synthetase, and deoxycytidine monophosphate deaminase [114]. Compared to other anticancer drugs, pancreatic cells exhibit more susceptibility towards gemcitabine treatment, and its use also enhances the quality of life factors for patients. However, the major drawback reported is that survival is prolonged for just one month [115]. Nevertheless, certain other regimens with the potential to improve the clinical efficiency of gemcitabine have also been documented [116]. Combination therapy with gemcitabine has given inconsistent results in clinical trials, and therefore extensive studies are warranted.

Chemo-resistance as a major hurdle in the treatment of pancreatic cancer has gained increased attention in recent years. It can arise from physiological barriers that prevent drug absorption or penetration into target tissues, or from biological mechanisms within individual tumor cells that reduce drug effectiveness at the intended site of action. Biological chemoresistance appears to arise as the result of three different types of mechanisms: 1), target cells can develop resistance to drug uptake; 2) target cells can develop altered sensitivity to drugs at their intended targets through, for example, increased expression of anti-apoptotic proteins; and 3) target cells can develop increased efflux of drugs, preventing drugs from reaching their intended site of action [117]. However, several recent studies have shown some encouraging outcomes using liposomes, nanoparticles and nanotubes mediated effective drug delivery that specifically target tumor microenvironment. Since desmoplasia as well as ECM components (including collagens I, III IV and etc) determine chemoresistance in pancreatic cancer cells, use of nanoparticles which can affect immune cells response within the tumor microenvironment are promising. The functional aspects of some of the available nano-based drug delivery against pancreatic cancer progression is discussed in Table 2. Even though few of these strategies have made it to clinical trials, the mechanisms by which desmoplasia support the tumor cells by offering resistance to drugs remains unclear and is the major obstacle in the anticancer therapies. Such research approaches to discover the associations of tumor cells with scar tissue around cancer cells, tumors, and surrounding stroma is highly warranted in the fight against pancreatic cancer, and is the research focus of our laboratory.

**Table 2:** Nano-based drug delivery system targeting tumor microenvironment for pancreatic cancer therapy

Compound	Mechanism of action	Ref
Gold nanoparticle	Inhibits proliferation and migration of pancreatic cancer cells and pancreatic stellate cells by disrupting the bidirectional communication; inhibit matrix deposition, enhance angiogenesis, and inhibit tumor growth	[142]
GDC-0449 + polymeric prodrug-based nanoparticles	Reduces fibroblast mediated drug resistance; Specifically reduce collagen, α-SMA and glioma-associated protein1 (GLI-1) expression in tumor tissues	[143]
Methacrylate-based Gemcitabine-monomer conjugate	Sustained release of Gemcitabine and enhances cytotoxicity against tumor growth	[144]
3,4-Difluorobenzylidene curcumin + styrene-maleic acid copolymer	Efficient intracellular delivery of 3,4-Difluorobenzylidene curcumin that specifically target tumor microenvironment	[145]
Bisnaphthalimidopropyldi-amino-octane + CH (5)-PAA polymer	Reduces tumor cell proliferation and tumor growth by inducing apoptosis	[146]
Gemcitabine + Hybrid nanoparticle	Systemic and targeted delivery of Gemcitabine to reduce tumor growth	[147]
Hybrid iron oxide-gold nanoparticles	Targeted drug delivery for tumor retardation	[148]
Paclitaxel + nanoemulsion	Enhances chemotherapeutic efficacy of drug by targeted delivery	[149]

### Current clinical trials targeting tumor-stroma in pancreatic cancer

A better understanding of the tumor microenvironment could lead to the development of novel therapeutic strategies against the stromal region of PDAC. The interaction of heterogeneous cancer cells with stromal compartments is suggested as the fundamental mechanism by which the neoplastic cells evade the cytotoxic effect of chemotherapeutic drugs [118]. In this context, manipulation of such cellular interactions may improve the therapeutic efficacy of drugs that reduce tumor metastasis and maintaining normal cellular functions. The preclinical evaluation of the efficacy of most of the drugs against PDAC have highly relied on in vitro analysis and in vivo experimental models. Several such studies have reported the effects of stromal manipulation and targeting desmoplasia in primary tumors as well as metastasis [119–121]. Interestingly, the ability of certain anti-fibrotic agents that selectively target stromal region to normalize the tumor microenvironment in PDAC has been reported. Pirfenidone [5-methyl-1-2-[1H]-pyridone], an anti-fibrotic drug used for the treatment of pulmonary fibrosis was reported as a promising anti-tumor agent for PDAC through regulating pancreatic stellate cells and suppression of desmoplastic reactions in a preclinical study [122]. Also, Ormeloxifene (ORM), a non-hormonal non-steroidal compound depletes tumor-associated stromal tissue by inhibiting the sonic hedgehog (SHH) signaling pathway in PDAC has been reported [123]. In another study it was reported that, depletion of hyaluronan, a non-sulphated glycosaminoglycan present in the ECM by PEGPH20, a PEGylated human recombinant PH20 hyaluronidase improves the drug delivery and responses in patients with pancreatic cancer. Further, the combination therapy of PEGPH20 and gemcitabine inhibited pancreatic tumor growth and prolonged survival of patients [121]. PEGPH20 is currently being tested in combination with nab-paclitaxel and gemcitabine in patients with stage IV untreated pancreatic cancer in its phase II clinical trials [124]. Several plants derived secondary metabolites are also reported to have anti-tumor efficacy against PDAC targeting desmoplastic reactions. Ellagic acid, a polyphenol found in fruits and nuts inhibited the cellular function and activation of pancreatic stellate cells through the suppression of platelet-derived growth factor (PDGF)-BB-induced tyrosine phosphorylation of β-receptor and the downstream activation of ERK and Akt signaling [125]. A novel curcumin-loaded magnetic nanoparticle was shown to have potent cancer prevention activity against PDAC through the depletion of collagen 1 and cell surface-associated mucin 1 (MUC1) [126]. In addition, a mathematical approach in pancreatic cancer study have revealed that PDAC do not progress in a linear manner always but can be result of simultaneous addition of genetically altered cells. In this point of view, testing of agents that target desmoplastic reactions in experimental models with localized primary tumors as well as metastatic tumors could be beneficial [127]. Apart from these pre-clinical studies, several clinical research attempts suggest that combination of drugs that target ECM and tumor associated stroma might be useful for identifying the beneficial of patients and for prolonging survival of patients (Table 3).

**Table 3:** Drugs in clinical trials for PDAC targeting cancer associated stroma

Compound	Combination	Target	Stage of patients	Trial phase	Ref	Outcome
Vismodegib	Gemcitabine	Hedgehog signaling	Stage IV PDAC	Phase I/II	[150]	Non- significant [151]
PEGPH20	Gemcitabine, nab-paclitaxel,	Hyaluronic acid	Stage IV PDAC	Phase II	[124]	Ongoing
EF-002	Dose escalation	Activity of macrophages	Solid tumor	Phase I	[152]	Ongoing
Paricalcitol	Gemcitabine, nab-paclitaxel	metabolic pathway	Advanced PC	Phase I	[153]	Ongoing
MEDI4736	Gemcitabine, nab-paclitaxel	C-X-C chemokine receptor type 2	Stage IV PDAC	Phase I/II	[154]	Ongoing
Defactinib	PD-1	FAK signaling	Solid tumor	Phase I/II	[155]	Ongoing
GSK2256098	Tremetinib	FAK signaling	PDAC	Phase II	[156]	Ongoing
retinoic acid	Gemcitabine, nab-paclitaxel	Cancer associated fibroblasts	PDAC	Phase I	[157]	Ongoing
AM0010	FOLFOX, 5-FU, leucovorin	Interleukins-10	Solid tumor	Phase III	[158]	Ongoing
Pembrolizumab	Paricalcitol, gemcitabine, nabpaclitaxel	PD-1	Resectable PC	Phase I	[159]	Ongoing
Cabiralizumab	Nivolumab	Colony-stimulating factor-1 receptor	Solid tumor	Phase I	[160]	Ongoing
Sonidegib	Gemcitabine, nab-paclitaxel	Hedgehog signaling	Stage IV PDAC	Phase I/II	[161]	Completed, Data not provided

Chemical compounds and combinations designed to target the specific desmoplastic features of pancreatic cancer offer new avenues for the development of stromal-based therapies. Manipulation of the tumor stromal compartments help in depriving cancer cells without affecting normal tissue functions. Together, these clinical approaches could offer significant insights into tumor-stroma interactions which is pivotal in tumor progression and could lead to the development of novel cancer associated desmoplasia-targeting agents for the treatment of pancreatic cancer.

## Conclusions

Cancer-associated desmoplasia and fibrotic response is a diverse and complex entity with widespread effects. The molecular mechanisms in these components are greatly interdependent, together shaping solid tumor formation and metastasis in the pancreas. Although outcomes in clinical trials for PDAC have not improved sufficiently in the recent decades, research attempts left a wealth of knowledge and insight which it is hoped that can be translated to achieve greater therapeutic success in the treatment of PDAC. Available reports propose the possibility of targeting specific ECM components in desmoplasia to prevent switching of cells within the tumorigenic and metastatic environment. This novel approach of targeting tumor desmoplasia and related mechanisms such as EMT could contribute to increased sensitivity of cancer cells to chemotherapy. By answering the complex research questions in and around tumor desmoplasia that remain obscure, we will be a step closer to the goal of diminishing patient suffering and improving overall prognosis and morbidity in PDAC.

## Abbreviations

CAF: Cancer-associated fibroblasts
ECM: Extracellular matrix
EMT: Epithelial-to-mesenchymal transition
MET: Mesenchymal-to-epithelial transition
PDAC: Pancreatic Ductal Adenocarcinoma
PDGF: Platelet derived growth factor
PSC: Pancreatic stellate cells
TGF β: Transforming growth factor beta
TLR: Toll-like receptor
VEGF: Vascular endothelial growth factor
α-SMA: Alpha smooth muscle actin
